# A novel germline mutation (c.A527G) in *STK11* gene causes Peutz–Jeghers syndrome in a Chinese girl: A case report

**DOI:** 10.1097/MD.0000000000008591

**Published:** 2017-12-08

**Authors:** Zi-Ye Zhao, Yu-Liang Jiang, Bai-Rong Li, Fu Yang, Jing Li, Xiao-Wei Jin, Shu-Han Sun, Shou-Bin Ning

**Affiliations:** a Department of Medical Genetics, Naval Medical University, Shanghai 200433, China; b Hebei North University, Zhangjiakou, Hebei Province, China; c Department of Gastroenterology, Airforce General Hospital of PLA, Beijing, China.

**Keywords:** hamartoma, Peutz–Jeghers syndrome, polyposis, *STK11* gene

## Abstract

**Rationale:**

Peutz–Jeghers syndrome (PJS) is a Mendelian autosomal dominant disease caused by mutations in the tumor suppressor gene, serine/threonine kinase 11 (STK11). The features of this syndrome include gastrointestinal (GI) hamartomas, melanin spots on the lips and the extremities, and an increased risk of developing cancer. Early onset of disease is often characterized by mucocutaneous pigmentation and intussusception due to GI polyps in childhood.

**Patient concerns:**

A girl with a positive family history grew oral pigmentation at 1 and got intussusception by small bowel hamartomas at 5.

**Diagnoses:**

She was diagnosed with PJS based on oral pigmentation and a positive family history of PJS.

**Interventions:**

Enteroscopy was employed to treat the GI polyps. Sanger sequencing was used to investigate STK11 mutation in this family.

**Outcomes:**

A large jejunal polyp together with other smaller ones was resected, and the girl recovered uneventfully. We discovered a heterozygous substitution in STK11, c.A527G in exon 4, in the girl and her father who was also a PJS patient, and the amine acid change was an aspartic acid-glycine substitution in codon 176. This mutation was not found in other healthy family members and 50 unrelated non-PJS controls, and it is not recorded in databases, which prove it a novel mutation. Evolutionary conservation analysis of amino acid residues showed this aspartic acid is a conserved one between species, and protein structure prediction by SWISS-MODEL indicated an obvious change in local structure. In addition, PolyPhen-2 score for this mutation is 1, which indicates it probably damaging.

**Lessons:**

PJS can cause severe complication like intussusception in young children, and early screening for small bowel may be beneficial for these patients. The mutation of STK11 found in this girl is a novel one, which enlarges the spectrum of STK11. Our analysis supported it a causative one in PJS.

## 1 Introduction

Peutz–Jeghers syndrome (PJS, OMIM 175200) is an autosomal dominant disorder characterized by gastrointestinal (GI) hamartomatous polyps, mucocutaneous pigmentation, and an increased risk for the development of GI and various extra-GI malignancies.^[1]^ Germline mutations in serine/threonine kinase 11 (*STK11*) gene are considered to cause PJS, and more than 400 mutations in it (Human Gene Mutation Database [HGMD], http://www.hgmd.cf.ac.uk) have been identified in patients with PJS. Though most reported cases of PJS were adults, over 30% of patients are younger than 10.^[2]^ The prominent characteristics of PJS in childhood are mucocutaneous pigmentation and intussusception due to GI polyps, and PJS-associated tumors are not many but do exist.^[3]^

Here, we report a 5-year-old PJS girl characterized by mucocutaneous pigmentation and small bowel polyps, and sequencing confirms the causative mutation is a novel one in *STK11*.

## 2 Case history

**Figure 1:**
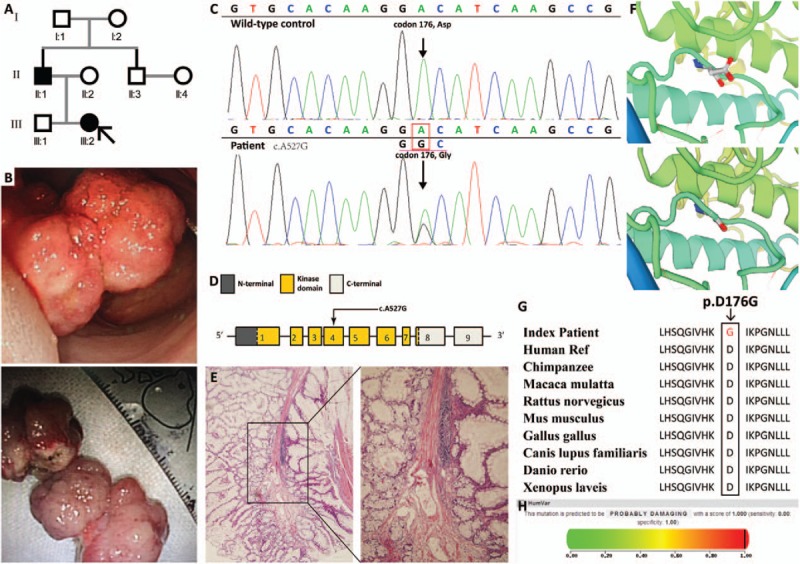
(A) The genogram of the proband. Roman numerals indicate generations and Arabic numbers indicate individuals. Squares = males, circles = females. Affected individuals are denoted by solid symbols and unaffected individuals are denoted by open symbols. The index patient is indicated by an arrow. (B) Endoscopic view of the largest polyp within jejunum. Up, in vivo; low, after piecemeal resection. (C) Sanger sequencing revealed a heterozygous substitution mutation, c.A527G. (D) The structure of *STK11* gene. This novel mutation is within exon 4. (E) Representative hematoxylin-eosin-stained tissue slices of the GI polyp specimens confirm hamartomatous. Left, ×40 magnification; right, ×100 magnification. (F) Local structure around the mutation site of the wild-type and mutant STK11 protein generated by SWISS-MODEL online software shows obvious difference. (G) Evolutionary conservation of amino acid residues altered by c. A527G (p. D176G) across different species. (H) PolyPhen-2 score for this mutation is 1, which indicates it probably damaging. GI = gastrointestinal, *STK11* = serine/threonine kinase 11.

The girl from Beijing southern suburb, whose father was a PJS patient, came to our department first time when she was 5 years old (Fig. 1A). Mucocutaneous pigmentation may become pronounced in lip and cheek when she was 1 year old, and her parents did not send her to hospital since no other symptoms occurred. Paroxysmal abdominal cramps and melena happened to her at her age of 5 just before she was referred to a children's hospital. CT scan revealed intussusception in the ileum, then the doctors there gave her air enema treatment, after which the cramps relieved to a great extent. Considering the symptoms (mucocutaneous pigmentation and intussusception) and her family history, they highly suspected the diagnosis of PJS and referred her to our department for further intervention in July 2015. Multiple small intestinal and colonic polyps, within which the diameter of the biggest one in proximal jejunum was 5 cm (Fig. 1B), were found and resected by enteroscopy, and pathological examination confirmed them hamartomas (Fig. 1E). The latest follow-up in September 2016 discovered no polyps within her small bowel by enteroscopy. All procedures involving human participants were in accordance with the ethical standards of the Medical Ethics Committee, Air Force General Hospital of PLA and with the 1964 Helsinki Declaration and its later amendments or comparable ethical standards.

## 3 Mutation analysis

During the proband's latest stay in hospital, we recruited her in the PJS surveillance and research program, and the blood samples of her and her available family members (I:1, I:2, II:1, II:2, II:3, and III:1) were collected (Fig. 1A). Genomic DNA of peripheral blood leucocytes was extracted routinely by Isolation Kit (DP318, Tiangen Biotech, Beijing, China) according to the manufacturer's instructions. All 9 coding exons of the *STK11* gene were amplified. PCRs of STK11 exons were performed in a 50-μL reaction which contained 0.4 μM of each primer, 50 ng genomic DNA, and 25 μL 2× premix Ex Taq DNA polymerase (RR030A, Takara Bio Inc., Dalian, China). The PCRs were performed under the following conditions: denaturation at 95°C for 4 minutes, followed by 35 thermal cycles, each composed of 95°C for 30 seconds, 58°C for 30 seconds, and 72°C for 45 seconds.

The index patient and available family members (relatives I:1, I:2, II:1, II:2, II:3, and III:1) underwent STK11 germline mutation testing to confirm cosegregation of the mutation with the disease. In order to rule out polymorphisms, 100 chromosomes from 50 unrelated control individuals who came to our department for gastric polyps in September 2016 were also screened for the presence of the mutation. All biological samples were collected after consent acquired

The PCR products were gel- and column purified and directly sequenced. The purified PCR fragments were then sequenced using BigDye Terminator (Applied Biosystems, Foster City, CA) on an ABI Prism 3100 genetic analyzer (Applied Biosystems, Foster City, CA) by Majorbio Co. Ltd. (Shanghai, China). The results were used to performance sequence alignment according to *STK11* gene sequence (NP_000446.1 and NM_000455.4 in GRCh38.p7). All experiment details and the primers used for PCR were reported previously.^[4,5]^

## 4 Mutation functional effect prediction, amino acid residues evolutionary conservation analysis and mutant protein structure prediction

Functional effect prediction of the mutation was carried out by the online tool PolyPhen-2 (http://genetics.bwh.harvard.edu/pph2/).

Evolutionary conservation of amino acid residues altered was analyzed by comparing across different species (https://www.ncbi.nlm.nih.gov/protein/STK11). The homology modeling programs, SWISS-MODEL (http://swissmodel.expasy.org), was used to develop an appropriate model to mimic the effects of the mutated region.^[6]^

## 5 Results

A survey of *STK11* gene revealed a heterozygous germline c.A527G mutation in exon 4 in the samples of the proband (III:2) and her father (II:1) (Fig. 1C and D), while this mutation was not detected in other available family members (I:1, I:2, II:2, II:3, and III:1) or the 50 unrelated control individuals. This mutation has not been reported in literatures or recorded in mutation databases such as dbSNP, ClinVar, and HGMD. This mutation resulted in an amine acid residue substitution, p.D176G. Evolutionary conservation analysis of amino acid residues showed this aspartic acid is a conserved one between species (Fig. 1G), and protein structure prediction by SWISS-MODEL presented the amine acid residue substitution caused an obvious change in local structure (Fig. 1F). In addition, PolyPhen-2 score for this mutation is 1, which indicates it probably damaging (Fig. 1H).

## 6 Discussion

Here we revealed a novel mutation in *STK11* causing PJS in a 5-year-old girl and her father. The mutation was only found in the PJS patients, but not in other healthy relatives or 50 unrelated control individuals. Since the mutation clearly co-segregates with the disease phenotype in the family, and structure and function prediction present the pathological effect of it, we conclude that this mutation is disease-specific and not a polymorphic variant of the *STK11* gene.

PJS first reported in the literature by Dr Connor published in 1895, and it adopted the current name in 1954 by Dr Bruwer.^[7]^ The pathogenic gene was cloned in 1997, which is *STK11* (OMIM 602216).^[8]^ The gene encodes a 433-amino-acid-residue protein, which acts as a tumor suppressor. By combined application of sanger sequencing and multiplex ligation-dependent probe amplification (MLPA), mutation detection rates have arisen to over 60% in most instances.^[9]^ Though frameshift or nonsense mutations are most common types, large defection and missense mutation are also disease causing. The STK11 protein is mainly comprised of an N-terminal noncatalytic domain, a catalytic kinase domain, and a C-terminal noncatalytic regulatory domain.^[10]^ Among reported *STK11* mutations (HGMD), most variants are located in the region of catalytic kinase domain (amino acids 49–309) and result in the absence of kinase activity and disrupting the formation of a complex to maintain kinase activation.^[11]^ So this novel mutation broaden the pathological mutation spectrum of *STK11* gene. It much likely results in a key structure change causing kinase activity impaired, which contributes to PJS phenotype.

When lacking a family history, PJS is often diagnosed when the patient suffers from intestinal obstruction and comes to the surgical emergency.^[12]^ With the widely use of endoscopy, PJS patients can receive intensively surveillance and largely avoid open surgery. Based on recommendation, people with a positive family history should receive first upper GI endoscopy and colonoscopy at age 8 years.^[13]^ More important, double-balloon enteroscopy (DBE) is the key method to detect and remove small bowel polyps nonoperatively.^[14]^ 131 PJS patients who had abdominal surgery for intestinal obstruction before have admitted to our hospital, and with the help of DBE, 113 of them (86.3%) avoid the second open surgery, indicating proper follow-up ensures the patients free of intestinal obstruction and malignancies.^[15]^ But in this case, the proband got intussusception at the age of 5, which indicated a more aggressive strategy may be necessary. The main risk of PJS in childhood and adolescence is the growth of GI polyps, not tumorigenesis. In our experience, child and teenager patients need more frequent surveillance than adult patients because of more rapid growth of GI polyps. So we suggest pay more attention to the surveillence for these patients.

## Acknowledgments

We thank the subjects for their participation. We appreciate very much for Dr Wen-Sheng Lin and Dr Hong-Yu Cheng's kindly help with the pathologic and endoscopic pictures of the polyps.
